# Integration of UPR^ER^ and Oxidative Stress Signaling in the Control of Intestinal Stem Cell Proliferation

**DOI:** 10.1371/journal.pgen.1004568

**Published:** 2014-08-28

**Authors:** Lifen Wang, Xiankun Zeng, Hyung Don Ryoo, Heinrich Jasper

**Affiliations:** 1 Buck Institute for Research on Aging, Novato, California, United States of America; 2 Basic Research Laboratory, Center for Cancer Research, National Cancer Institute, Frederick, Maryland, United States of America; 3 Department of Cell Biology, New York University School of Medicine, New York, New York, United States of America; Harvard Medical School, Howard Hughes Medical Institute, United States of America

## Abstract

The Unfolded Protein Response of the endoplasmic reticulum (UPR^ER^) controls proteostasis by adjusting the protein folding capacity of the ER to environmental and cell-intrinsic conditions. In metazoans, loss of proteostasis results in degenerative and proliferative diseases and cancers. The cellular and molecular mechanisms causing these phenotypes remain poorly understood. Here we show that the UPR^ER^ is a critical regulator of intestinal stem cell (ISC) quiescence in *Drosophila* *melanogaster.* We find that ISCs require activation of the UPR^ER^ for regenerative responses, but that a tissue-wide increase in ER stress triggers ISC hyperproliferation and epithelial dysplasia in aging animals. These effects are mediated by ISC-specific redox signaling through Jun-N-terminal Kinase (JNK) and the transcription factor CncC. Our results identify a signaling network of proteostatic and oxidative stress responses that regulates ISC function and regenerative homeostasis in the intestinal epithelium.

## Author Summary

Loss of proper protein homeostasis (proteostasis) as well as increased production of reactive oxygen species (ROS) is a hallmark of aging. In complex metazoans, these processes can result in proliferative diseases and cancers. The protein folding capacity of the endoplasmic reticulum (ER) is monitored and maintained by the unfolded protein response of the ER (UPR^ER^). In this study, we identify a coordinated role of UPR^ER^ and oxidative stress signaling in regulating the proliferation of intestinal stem cells (ISCs). We find that the ER-stress responsive transcription factor Xbp1 and the ER-associated degradation pathway component Hrd1 are sufficient and required cell autonomously in ISCs to limit their proliferative activity. This function is dependent on the activities of the stress sensor JNK and the redox-responsive transcription factor CncC, which we have previously identified as regulators of ISC proliferation. We further show here that promoting ER homeostasis in aging ISCs is sufficient to limit age-associated epithelial dysplasia. Our results establish the integration of UPR^ER^ and oxidative stress signaling as a central mechanism promoting regenerative homeostasis in the intestinal epithelium.

## Introduction

Long-term homeostasis of high-turnover tissues relies on the precise regulation of stem cell (SC) activity that allows tailoring regenerative responses to the needs of the tissue. Regenerative processes in barrier epithelia, such as the intestinal epithelium, are particularly vulnerable to exogenous insults. Understanding how cellular stress responses of intestinal epithelial cells (IECs) and intestinal stem cells (ISCs) coordinate and maintain regenerative processes in the gut will provide insight into the etiology of pathologies ranging from inflammatory bowel diseases (IBDs) to colorectal cancers.

The unfolded protein response of the ER (UPR^ER^) plays a central role in the control of homeostasis of the intestinal epithelium. Loss of protein folding capacity in the ER of IECs results in complex cell-autonomous and non-autonomous activation of stress signaling pathways, triggering an inflammatory condition that severely perturbs proliferative homeostasis, innate immune function and cell survival in the epithelium, and has been implicated in IBDs [1]–[7].

**Figure 1:**
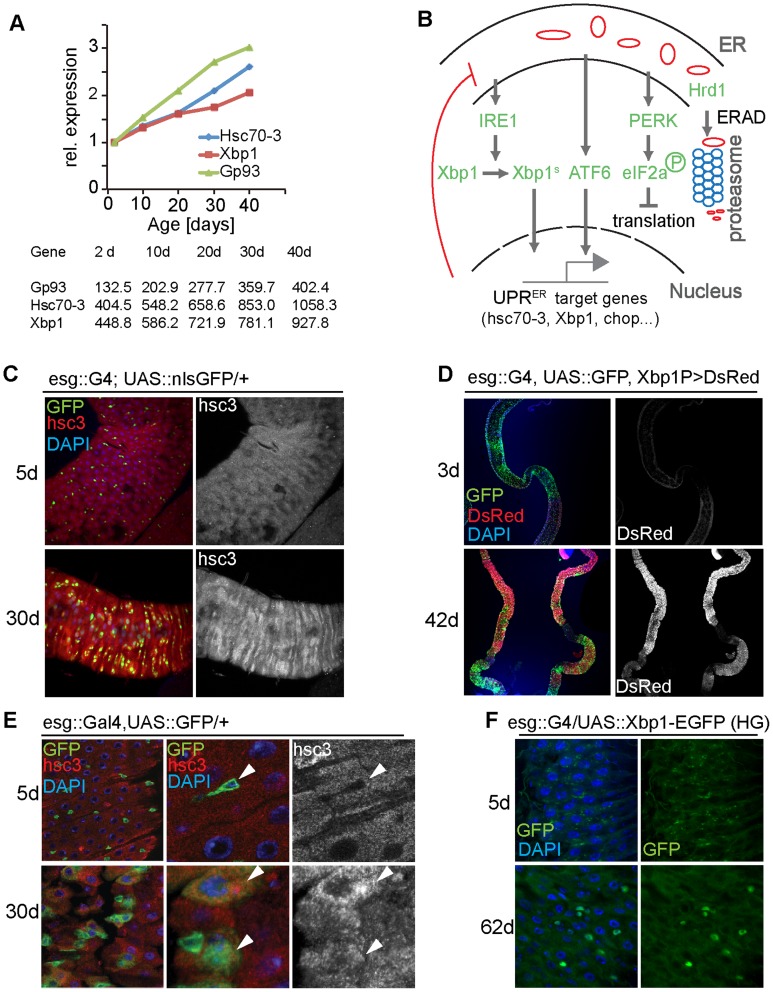
The UPR^ER^ is activated in aging intestines. (A) Expression of Gp93, Hsc70-3 and Xbp1 in guts from flies of different ages. Expression was determined using RNAseq in experiments described in [32]. Expression values are shown normalized to the 2 d timepoint in the graph. Raw RPKM values are shown in the table. (B) Major branches of UPR^ER^ signaling pathways in *Drosophila*. (C) Young (5 day old) and old (30 day old) guts of esg::Gal4, UAS::GFP flies immunostained with anti-Hsc3 antibody (DNA: DAPI, blue; ISCs/EBs: GFP, green; Hsc3, red). The Hsc3 channel is shown separately on the right. (D) Reporter line *Xbp1_p_*::*DsRed* visualizes expression of Xbp1 in young (3 day) and old (42 day) guts (DNA: DAPI, blue; ISCs/EBs: GFP, green; DsRed: red). The DsRed channel is shown separately on the right. (E) Enlarged images for intestines of young (5 day) and old (30 day) flies (esg::Gal4, UAS:: GFP/+) immunostained with anti-Hsc3 antibody (DNA: DAPI, blue; ISCs/EBs: GFP, green; Hsc3, red). White arrowheads indicate ISCs/EBs. The Hsc3 channel is shown separately on the right. (F) Xbp1 splicing reporter (UAS::Xbp1-EGFP) expressed in ISCs/EBs using esg::Gal4. Expression of GFP in young (5 day) and old (62 day) guts is shown. (DNA: DAPI, blue; GFP, green). The GFP channel is shown separately on the right. See also Figure S1.

The UPR^ER^ is triggered by the accumulation of misfolded proteins in the ER [8], which activate three highly conserved UPR^ER^ sensors: the PKR-like ER kinase PERK, the transcription factor ATF6, and the endoribonuclease IRE1 (Figure 1B). These sensors make up the three branches of UPR^ER^ signaling, which consists of IRE1-mediated splicing of the mRNA encoding the bZip transcription factor X-Box binding protein 1 (Xbp1), phosphorylation of the translation initiation factor 2 alpha (eIF2α) by PERK, and cleavage and activation of ATF6, resulting in its nuclear translocation and activation of stress response genes, including Xbp1 [1]–[7], [9]. Xbp1 regulates transcription of ER components, and the resulting transcriptional induction of ER chaperones and of genes encoding ER components enhances ER folding capacity, and the reduction in protein synthesis (by eIF2α) alleviates the protein load in the ER. Furthermore, factors required to degrade un/misfolded proteins through ER-associated degradation (ERAD) are induced [8], [10]–[12]. The accumulation of un/misfolded proteins in the ER is further associated with increased production of reactive oxygen species (ROS), most likely due to the production of hydrogen peroxide as a byproduct of protein disulfide bond formation by protein disulfide isomerase (PDI) and ER oxidoreductin 1 (Ero1) [13]–[15].

Recent studies suggest that the UPR^ER^ may influence regenerative processes in the gut directly, as it is engaged in cells transitioning from a stem-like state into the transit amplifying state in the small intestine of mice [16]. Regeneration is also influenced by the intracellular redox state of stem cells, and changes in intracellular ROS production play an important role in the regulation of SC pluripotency, proliferative activity, and differentiation [17]–[20]. Coordinated control of cellular protein and redox homeostasis by the UPR^ER^ and other stress signaling pathways is therefore critical to maintain SC function. Exogenous ER stress likely disrupts this coordination, perturbing regeneration and proliferative homeostasis. Consistent with this model, excessive UPR^ER^ activity has been implicated in tumorigenesis [2], [21].

To understand the long-term maintenance of epithelial homeostasis in the intestine, detailed insight into the regulation and function of the UPR^ER^ and its coordination with the redox response in the intestinal epithelium, in a cell-type specific and temporally resolved manner, is required. Here, we have initiated such an analysis, using the *Drosophila* intestinal epithelium as a model system. The *Drosophila* ISC lineage exhibits a high degree of functional and morphological similarities with the ISC lineage in the mammalian small intestine [22]–[24]. When a regenerative response is induced in the intestinal epithelium, ISCs self-renew and give rise to transient, non-dividing progenitor cells called EnteroBlasts (EBs), which differentiate into either absorptive EnteroCytes (ECs) or secretory EnteroEndocrine (EEs) cells, triggered by differential Notch signaling. ISCs are the only dividing cells in the posterior midgut of *Drosophila* and their entry into a highly proliferative state is regulated by multiple stress and mitogenic signaling pathways, including Jun-N-terminal Kinase (JNK), Jak/Stat, Insulin, Wnt, and EGFR signaling [24], [25]. The transcription factor CncC (orthologue of mammalian Nrf2 and worm SKN-1), a master regulator of intracellular redox homeostasis, controls proliferation of ISCs by limiting ROS accumulation [19]. Interestingly, mammalian Nrf2 has been suggested to buffer ROS production during ER stress, while worm SKN-1 has recently been found to coordinate antioxidant gene expression with Xbp1 [26], [27].

During aging, flies develop epithelial dysplasia in the intestine, caused by excessive ISC proliferation and deficient differentiation of EBs [28], [29]. This phenotype is a consequence of an inflammatory condition initiated by dysbiosis of the commensal bacteria, and causes metabolic decline, loss of epithelial barrier function, and increased mortality [30]–[32]. The ISC-intrinsic mechanisms causing the decline of proliferative homeostasis in the aging intestinal epithelium remain unclear.

Here, we have dissected the role of the UPR^ER^ and redox signaling in the control of ISC function and epithelial homeostasis at cellular resolution. We find that ER homeostasis is lost in the aging intestinal epithelium, and that this loss correlates with intestinal dysplasia. Activation of the UPR^ER^ within ISCs is required and sufficient for ISC proliferation, and excessive ER stress contributes to the age-associated dysplasia observed in the *Drosophila* gut. These effects are mediated by changes in the intracellular redox state, which perturb Nrf2/CncC and JNK activities. Accordingly, we find that JNK and Nrf2/CncC act epistatically in the control of ISC proliferation by ER stress. Our findings provide an integrated model for the regulation of ISC activity by redox and proteostatic signaling, and highlight the effects of this integration on epithelial homeostasis.

## Results

### UPR^ER^ activation in aging *Drosophila* intestines

In a recent transcriptome analysis of age-related changes in the *Drosophila* intestine [32], we noticed that expression of the ER stress-responsive genes *Bip/Hsc70-3/Hsc3*, the ER chaperone *Gp93*, and *Xbp1* are significantly induced in aging guts (Figure 1A). To confirm these findings, we used an antibody against Hsc3 and a reporter for Xbp1 expression, Xbp1_P_::dsRed [33], and assessed their expression in young and old guts (Figure 1C, 1D, S1H). Consistent with the RNAseq results, Hsc3 immunoreactivity and Xbp1 expression increased throughout the posterior midgut of aging flies (Figure 1C, 1D, S1H), suggesting that the UPR^ER^ is activated in the aging intestinal epithelium. Since ER stress has been implicated in deregulation of mammalian ISC function [2], [16], and since *Drosophila* ISCs over-proliferate in aging guts, causing epithelial dysplasia [28], [29], [32], we assessed whether the UPR^ER^ is activated in aging ISCs. ISCs and EBs can be identified in the posterior midgut of flies by expression of GFP driven by the esg::Gal4 driver, and ISCs can further be identified by expression of the Notch ligand Delta (DI). In young animals, Hsc3 was expressed at lower levels in progenitor cells than in differentiated cells. In old guts however, Hsc3 expression was strongly increased in progenitor cells, suggesting a specific activation of the UPR^ER^ in these cells (Figure 1E, Figure S1H). We confirmed this using an Xbp1 splicing reporter [11], [34], which assesses activation of Ire1. When this reporter was expressed in ISCs and EBs using esg::Gal4, no activity was detected in young flies, but GFP fluorescence was readily detectable in old guts (Figure 1F).

### Control of ISC proliferation by the UPR^ER^

**Figure 2:**
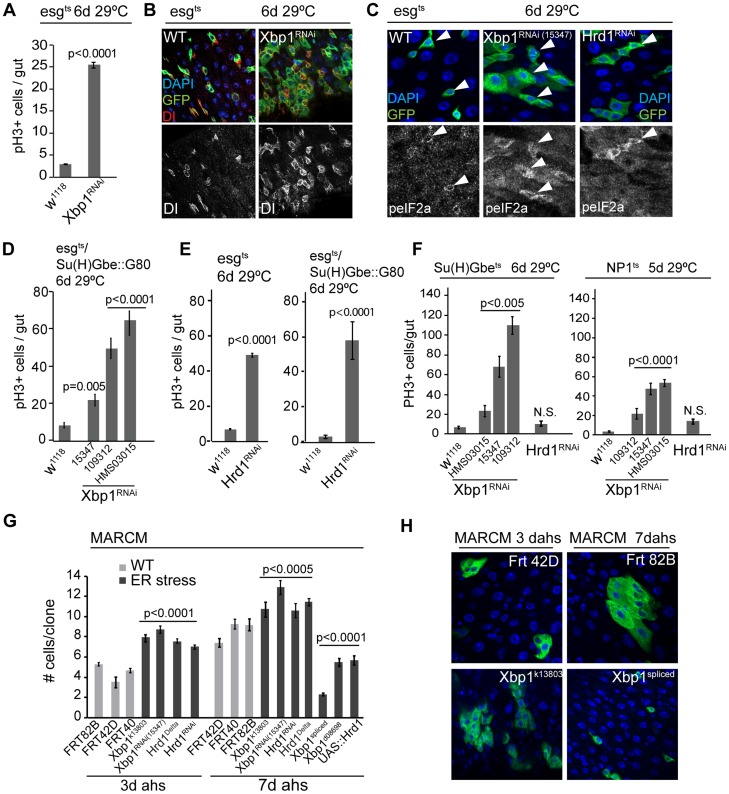
The UPR^ER^ is sufficient and required in ISCs to promote proliferation. (A) Knockdown of Xbp1 (using Xbp1RNAi^15347^) in ISCs/EBs (using esg::Gal4, tubG80^ts^) leads to ISC over-proliferation in intestines, quantified by counting the number of pH3+ cells/gut. (B) Representative images of wild-type flies and flies with ISC/EB-specific knockdown of Xbp1 (DNA: DAPI, blue; ISCs/EBs marker: GFP, green; ISC marker: DI, red/white). Dl channel is shown separately in lower panels. (C) Loss of Xbp1 or Hrd1 in ISCs/EBs promotes eIF2α phosphorylation in ISCs/EBs, compared to wild-type control. peIF2α channel is shown in white in lower panels. Arrowheads for orientation. DNA: DAPI, blue; ISCs/EBs: GFP, green; peIF2α, grey. (D) ISC-specific knockdown of Xbp1 (using esg::Gal4, Su(H)-Gbe::G80,tub::Gal80ts) induces ISC proliferation. 3 different fly lines expressing dsRNA against Xbp1 in a Gal4-sensitive manner (Xbp1RNAi^15347^, Xbp1RNAi^109312^, and Xbp1RNAi^HMS03015^) were used. Averages and SEM are shown. P values from Student's T test, N>20. (E) Knockdown of Hrd1 in ISCs/EBs (using esg::Gal4, tubG80ts), or specifically in ISCs (esgts/Su(H)Gbe::G80) induces ISC proliferation. Averages and SEM are shown. P values from Student's T test, N>10. (F) Quantification of pH3^+^ cells in wild-type flies and in flies expressing RNAi constructs against Xbp1 or Hrd1 in EBs (using Su(H)Gbe::Gal4, tub::Gal80ts) or ECs (using NP1::Gal4, tub::Gal80ts). Averages and SEM are shown. P values from Student's T test, N = 10. (G) Quantification of MARCM clone sizes at 3 days and 7 days after heat shock for *Xbp1* and *hrd1* loss-of-function (Xbp1^k13803^, Xbp1^RNAi^, Hrd1^Delta^, Hrd1^RNAi^) or gain-of-function (Xbp1^spliced^, Xbp1^d08698^ and UAS::Hrd1) conditions. Averages and SEM are shown. P values from Student's T test. Number of clones examined: n = 447 (3d FRT82B); n = 42 (3d FRT42D); n = 165 (3d FRT40); n = 95(3d Xbp1^k13803^); n = 268 (3d Xbp1RNAi); n = 515 (3d Hrd1^Delta^); n = 262 (3d Hrd1RNAi); n = 178 (7d FRT42D); n = 215 (7d FRT40); n = 394 (7d FRT82B); n = 123 (7d Xbp1^k13803^); n = 270 (7d Xbp1RNAi); n = 424 (7dHrd1^Delta^); n = 119 (7d Hrd1RNAi); n = 99 (Xbp1^spliced^); n = 138 (Xbp1^d08698^); n = 162 (UAS::Hrd1). (H) Representative images for Xbp1 loss-of-function at 3 days after heat shock and spliced Xbp1 at 7 days after heat shock are shown on the right. (DAPI, blue; GFP, green). See also Figure S1.

To test whether the loss of ER homeostasis is a cause or a consequence of the age-associated over-proliferation of ISCs, we examined the proliferative activity of ISCs in which Xbp1 had been knocked down by RNAi (the effectiveness of the RNAi and over-expression constructs used here and below were validated by RT-PCR; Figure S2D). We used the esg::Gal4 driver in combination with tub::Gal80ts, which allows temperature-inducible expression of UAS-controlled dsRNAs in ISCs and EBs (this combination is labeled esg^ts^ throughout). Perturbing Xbp1 was sufficient to strongly induce ISC proliferation, as measured by the frequency of cells positive for the mitotic marker phospho-Histone H3 (pH3; Figure 2A), and by the expansion of Dl+/esg+ cells within the epithelium (Figure 2B).

We confirmed the induction of ISC proliferative activity in these conditions by assessing the rate of tissue turnover in flies in which Xbp1^RNAi^ and GFP were heritably expressed in ISCs and their daughter cells in response to an ISC-specific recombination event (using esg::Gal4, tubGal80ts, UAS::GFP, UASFlp, act>STOP>Gal4, also termed esg^ts^F/O, [35]). Knocking down Xbp1 greatly accelerated epithelial renewal compared to wild type conditions, further highlighting a role for ER stress in promoting ISC proliferation (Figure S1A). The over-proliferation induced by knocking down Xbp1 in ISCs also increased phosphorylation of eIF2α, which is phosphorylated in response to ER stress by PERK, indicating that this induction of ISC proliferation is associated with an activation of the UPR^ER^ (Figure 2C).

We asked whether the proliferative response of ISCs in Xbp1 loss of function conditions was induced by changes in ER homeostasis in ISCs specifically, or whether induction of ER stress in EBs or daughter cells was driving ISC proliferation non-autonomously. To address this question, we first restricted expression of Xbp1^RNAi^ to ISCs, by combining esg::Gal4 with a transgenic construct that inhibits Gal4 activity in EBs (Su(H)-Gbe::Gal80; Su(H)-Gbe promoter elements are activated specifically in EBs in response to Dl/N signaling in EBs [36]; Figure S2E). Using 3 different dsRNA constructs against Xbp1, we confirmed that Xbp1 knockdown specifically in ISCs is sufficient to induce ISC proliferation (Figure 2D, Figure S1G). Xbp1 has a complex role in ER homeostasis, serving both as a sensor for ER stress and as a promoter of ER growth and proteostasis [11], [34], [37], and may also act independently of the ER stress response to regulate ISC proliferation. We therefore tested if independently activating the UPR^ER^, by impairing the removal of unfolded proteins in the ER directly, is sufficient to induce ISC proliferation. To this end, we perturbed the ER-associated degradation (ERAD) pathway by knocking down the ERAD-associated E3 ubiquitin ligase Hrd1, which is required for ubiquitination and degradation of unfolded proteins in the ER [38]. Knockdown of Hrd1 also induced eIF2α phosphorylation in ISCs (Figure 2C) and increased ISC proliferation, both when driven by esg^ts^ and when driven by esg^ts^ in combination with Su (H)::Gal80 (Figure 2E, Figure S1G). We also examined whether knocking down Xbp1 or Hrd1 in other cell types of the gut epithelium is sufficient to promote ISC proliferation. Knocking down Xbp1, but not Hrd1 in EBs (using Su(H)Gbe::Gal4 [39] in combination with tub::Gal80ts) or in ECs (using NP1::Gal4, tub::Gal80ts) increased ISC proliferation (Figure 2F), suggesting the existence of an Xbp1-specific non-autonomous effect on ISC proliferation in this tissue. Taken together, our results indicate that loss of ER homeostasis within ISCs induces ISC proliferation. The non-autonomous feedback of Xbp1 perturbation in ISC daughter cells on ISC proliferative activity is interesting and will be explored mechanistically in a separate study (Wang et al., in preparation).

If activation of the UPR^ER^ is required for the regenerative response of ISCs, perturbation of UPR^ER^ components should influence the growth of ISC-derived cell clones. To test this idea, and to determine the requirement for UPR^ER^ components in the regulation of ISC activity in homeostatic conditions, we performed linage tracing of mutant stem cells via the MARCM system [40]. Clones generated by ISCs homozygous for the Xbp1 loss-of-function allele *Xbp1^k13803^*, the Hrd1 loss of function allele *hrd1^Delta^* (a deletion that deletes Hrd1 and CG2126, see methods), or clones expressing Xbp1^RNAi^ or Hrd1^RNAi^ grew significantly faster than wild-type controls (Figure 2G, Figure 2H, Figure S1B–E). Accordingly, clones derived from ISCs over-expressing endogenous Xbp1 (using Xbp1^d08698^, a line in which Xbp1 transcription is induced downstream of a transgenic UAS [37], [41]), a transgene encoding a constitutively active, spliced version of Xbp1 [11], or transgenic Hrd1 [42], grew significantly slower than clones derived from wild-type ISCs (Figure 2G, Figure 2H, Figure S1F). While maintaining ER homeostasis through the UPR^ER^ is thus essential to limit ISC proliferation and prevent dysplasia, a functional UPR^ER^ is also required for normal homeostatic regeneration.

### UPR^ER^ as a rheostat for stem cell proliferation

**Figure 3:**
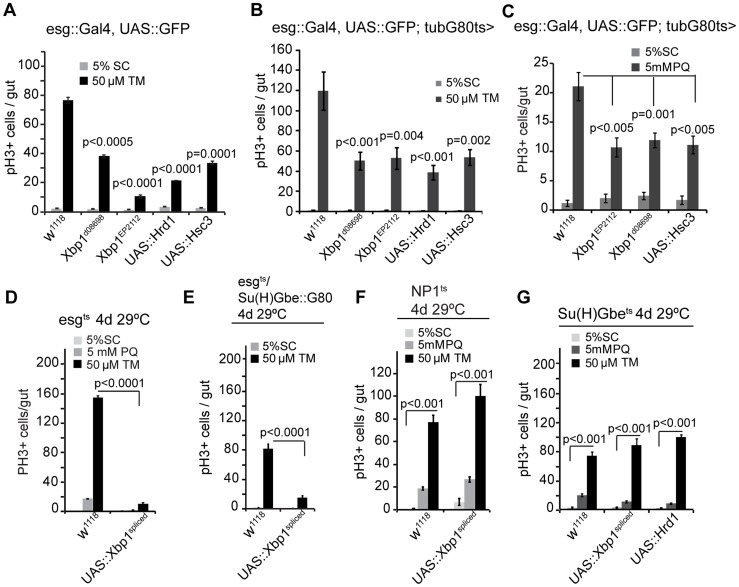
The UPR^ER^ as a rheostat for stem cell proliferation. (A) Quantification of pH3^+^ cells in wild-type flies and in flies over-expressing Xbp1 (*xbp1^d08698^*, *xbp1^EP2112^*), Hrd1, or Hsc3 under the control of esg::Gal4 exposed to mock treatment (5% sucrose) or tunicamycin (TM). Averages and SEM are shown. P values from Student's T test, N = 10. (B) Quantification of pH3^+^ cells in wild-type flies and in flies over-expressing Xbp1 (*Xbp1^d08698^*, *Xbp1^EP2112^*), Hrd1, or Hsc3 under the control of esg::Gal4, UAS::GFP; tub::Gal80^ts^ exposed to mock treatment (5% sucrose) or tunicamycin (TM). Averages and SEM are shown. P values from Student's T test, N = 10. (C) Quantification of pH3^+^ cells in wild-type flies and in flies over-expressing Xbp1 (*Xbp1^d08698^*, *Xbp1^EP2112^*), or Hsc3 under the control of esg::Gal4, UAS-GFP; tubG80^ts^ exposed to mock (5% sucrose) and oxidative-stress inducer paraquat (5 mM PQ). Averages and SEM are shown. P values from Student's Test, N = 10. (D) Spliced Xbp1 prevents ISC over-proliferation induced by excessive ER stress. Quantification of mitotic figures of wild-type flies and flies over-expressing spliced Xbp1 (Xbp1^spliced^) in ISCs/EBs (esg::Gal4, UAS-GFP; tubG80^ts^) exposed to mock treatment (5% sucrose) or paraquat (5 mM) or tunicamycin(50 µM). Averages and SEM are shown. P values from Student's Test, N = 10. (E) Over-expressing spliced Xbp1 in ISCs only (using esg::Gal4, Su(H)-Gbe::G80,tub::Gal80ts) inhibits stress-induced ISC proliferation. Averages and SEM are shown. P values from Student's T test, N>10. (F) Over-expressing spliced Xbp1 in ECs has no effect on ISC proliferation upon stress. Quantification of PH3^+^ cells in intestines of wild-type flies and in flies expressing Xbp1^spliced^ specifically in ECs (using NP1::Gal4; tub::Gal80^ts^). Averages and SEM are shown. P values from Student's T test, N = 10. (G) Quantification of pH3^+^ cells in intestines of wild-type flies and in flies expressing spliced Xbp1 (Xbp1^DSΔ28^) or Hrd1 specifically in EBs (using Su(H)Gbe::Gal4,tubG80^ts^) exposed to mock treatment (5% sucrose), paraquat (5 mM), or tunicamycin (50 µM). Averages and SEM are shown. P values from Student's Test, N = 10. See also Figure S1, S2.

To further confirm that promoting ER homeostasis within ISCs selectively limits their proliferation, we assessed if increasing the expression of UPR^ER^ components in ISCs or their daughter cells was sufficient to allay tunicamycin-induced ISC proliferation. Tunicamycin, potently induces ER stress by inhibiting N-linked protein glycosylation and thus impairing protein folding [43]. Feeding tunicamycin very robustly induced ISC proliferation, supporting a role for activation of the UPR^ER^ in promoting ISC proliferation (Figure 3A, Figure 3B, Figure 3D, and Figure 3E). Increasing ER stress tolerance by over-expressing endogenous Xbp1, spliced Xbp1, Hrd1, or Hsc3/Bip in ISCs and EBs (using esg::Gal4 and esg^ts^; spliced Xbp1 was expressed only in adults using esg^ts^) is sufficient to significantly reduce tunicamycin-induced ISC proliferation (Figure 3A, Figure 3B, Figure 3D, note that expressing spliced Xbp1, endogenous Xbp1 (using Xbp1^d08698^ or Xbp1^EP2112^ [37], [41]), as well as Hsc3/Bip also inhibited proliferation induced by oxidative stress inducer paraquat, Figure 3C, Figure 3D,). This inhibition was also observed when spliced Xbp1 was over-expressed selectively only in ISCs (using esg^ts^; Su(H)Gbe::Gal80; Figure 3E), but not when spliced Xbp1 or Hrd1 were expressed in ECs or EBs only (using the EC-specific NP1::Gal4 or the EB-specific Su(H)Gbe::Gal4, both rendered heat-inducible by combination with tub::Gal80ts; Figure 3F, Figure 3G). Altogether, our data indicate that maintaining ER homeostasis in ISCs is critical for long-term ISC quiescence, while an active UPR^ER^ within ISCs is required and sufficient for ISC proliferation under homeostatic conditions, as well as in response to ER or oxidative stress. To assess whether engaging the UPR^ER^ is universally required for ISC proliferation, we assessed if reducing ER stress by over-expressing spliced Xbp1 was sufficient to limit ISC proliferation in a range of mitogenic conditions. ISC proliferation can be triggered through the JNK or the insulin/IGF signaling (IIS) pathways by over-expressing the JNK Kinase Hemipterous (Hep) [28] or the Insulin Receptor (InR) [44]–[46]. Over-expression of spliced Xbp1 was sufficient to inhibit ISC proliferation in both conditions (Figure S2A, Figure S2B; this inhibition is not due to apoptosis of ISCs, as ISCs were readily observed even at 14 days after inducing expression of Hep and/or Xbp1^spliced^).

Modulating Xbp1 activity further influenced the growth of ISC/EE tumors that accumulate due to defective EB differentiation in Notch loss of function conditions: While spliced Xbp1 prevented tumor formation, loss of Xbp1 exacerbated the growth of these tumors (Figure S2C). By regulating ER homeostasis, Xbp1 thus serves as a rheostat broadly controlling ISC proliferative activity.

### ROS-mediated control of ISC proliferation by the UPR^ER^

**Figure 4:**
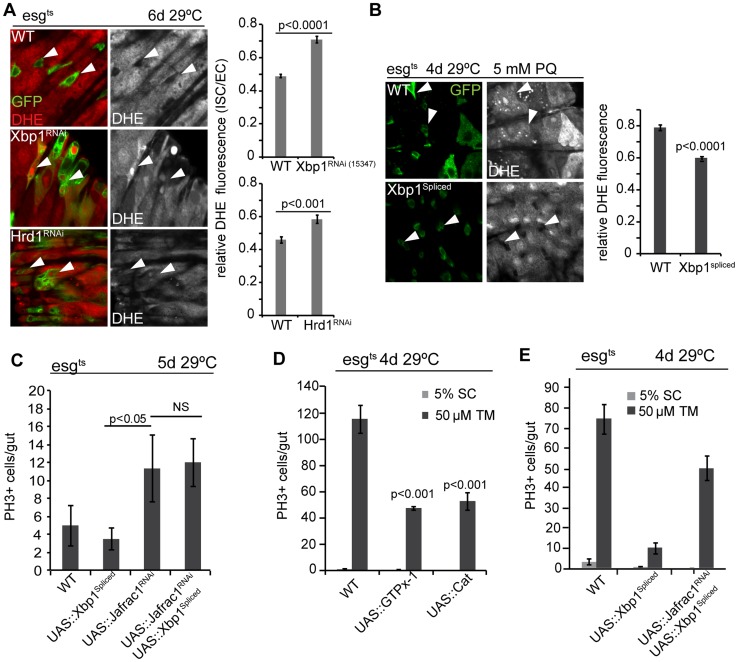
Coordinated control of ISC proliferation by the UPR^ER^ and ROS signaling. (A) Increased ROS in ISCs deficient in Xbp1 or Hrd1. Relative DHE fluorescence intensity is calculated by the ratio of fluorescence intensity in ISCs/EBs and nearby ECs. ISCs/EBs identified by GFP. Arrowheads point to selected ISCs/EBs. GFP, green; DHE red; DHE is shown as separate channel in white. Averages and SEM for relative DHE intensity are shown. P values from Student's T test, N>200 (from 6–7 WT, Xbp1^RNAi^ guts), N>100 (from 3–4 WT, Hrd1^RNAi^ guts). (B) Over-expression of spliced Xbp1in ISCs/EBs (using esg::Gal4, tubG80^ts^) resulted in decreased DHE fluorescence in ISCs under Paraquat treatment. Relative DHE fluorescence intensity is quantified for wild-type flies and flies over-expressing spliced Xbp1. Arrowheads point to selected ISCs/EBs. GFP: green. DHE is shown as separate channel in white. Averages and SEM are shown. P values from Student's Test. (C) Frequency of pH3+ cells are quantification for wild-type fly and flies expressing spliced Xbp1 only, Jafrac1 loss-of-function only, and for flies coexpressing spliced Xbp1 and Jafrac1 loss-of-function. Averages and SEM are shown. P values from Student's Test. N = 10. (D) Over-expression of anti-oxidant enzyme GTPx-1 or Cat in ISCs/EBs (esg::Gal4,tubG80ts) inhibits tunicamycin-induced ISC proliferation. Averages and SEM are shown. P values from Student's T test. N>10. (E) Quantification of pH3^+^ cells in wild-type flies, in flies over-expressing spliced Xbp1 only, and in flies expressing both Jafrac1 loss-of-function and spliced Xbp1 under the control of esg::Gal4, UAS-GFP; tubG80^ts^ exposed to mock (5% sucrose) and tunicamycin. Averages and SEM are shown. P values from Student's Test. N = 10.

The control of ISC proliferation by the UPR^ER^ resembles ISC control by ROS, which can trigger dysplastic over-proliferation of ISCs, but are required for proliferation during homeostatic regeneration [19], [24]. Oxidative stress and ER stress are tightly linked: perturbation of redox homeostasis results in the accumulation of misfolded proteins and activation of the UPR^ER^, and ER stress results in cytosolic oxidative stress [47]–[49]. To explore the relationship between ER stress, oxidative stress, and the UPR^ER^ in the ISC lineage, we assessed changes in intracellular redox homeostasis in ISCs deficient in Xbp1 or Hrd1 (Figure 4A). Both conditions resulted in significantly increased fluorescence of dihydro-ethidium (DHE, a redox-sensitive dye that can be used to detect ROS accumulation in live intestines [17], [19]) compared to wild-type progenitor cells (Figure 4A). Over-expression of spliced Xbp1, in turn, resulted in decreased DHE fluorescence in ISCs even under stress conditions (Figure 4B).

To further dissect the relationship between ER stress and oxidative stress, we perturbed the peroxiredoxin Jafrac1, which strongly influences intracellular redox homeostasis and regulates ISC proliferation [19], [50]. Knockdown of Jafrac1 was sufficient to increase ISC proliferation, and this increase was insensitive to the expression of spliced Xbp1 (Figure 4C). Over-expression of the anti-oxidant enzymes glutathione peroxidase I (GTPx-1) or Catalase (Cat), on the other hand, inhibited tunicamycin-induced ISC proliferation (Figure 4D), while knocking down Jafrac1 in ISCs prevented the inhibition of tunicamycin-induced ISC proliferation by spliced Xbp1 (Figure 4E). Increased ROS production thus acts downstream of Xbp1 in the regulation of ISC proliferation.

**Figure 5:**
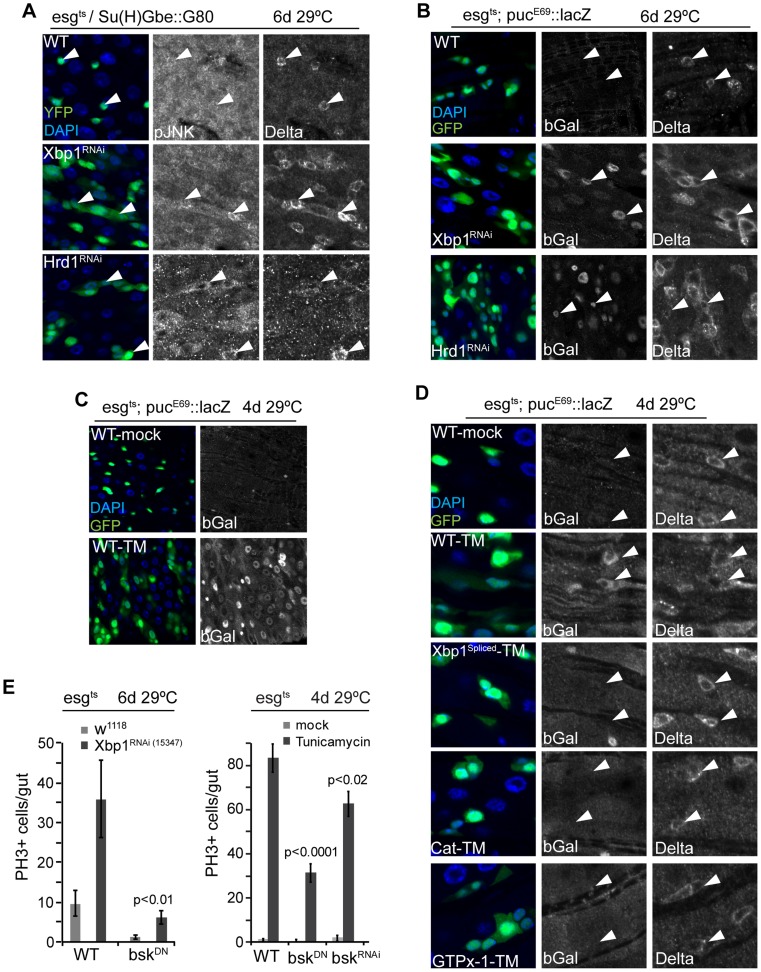
JNK is activated by UPR^ER^-induced ISC proliferation. (A) JNK is phosphorylated in ISCs when Xbp1 or Hrd1 is knocked down specifically in ISCs. DAPI, blue; GFP, green; pJNK or Dl shown as separate channels in white. (B) JNK activation when Xbp1 or Hrd1 is knocked down in ISCs/EBs. Anti bGal antibody staining to detect lacZ expression from the puc^E69^::lacZ reporter. DAPI, blue; GFP, green; bGal or DI is shown as separate channel in white. (C) JNK activation by tunicamycin exposure. Anti bGal antibody was used to detect expression of lacZ from a puc::lacZ reporter (esg::Gal4, UAS::GFP, tubG80ts/pucE69::lacZ). Flies were exposed to mock treatment (5% sucrose) or tunicamycin (50 µM in 5% sucrose). DNA: DAPI, blue; ISCs/EBs: GFP, green; bGal, white. (D) Over-expression of spliced Xbp1 or of GTPx-1 or Cat in ISCs/EBs (esg::Gal4,tubG80ts) represses tunicamycin-induced JNK activation. bGal antibody staining detecting lacZ expression from the puc^E69^::lacZ reporter. DNA: DAPI, blue; ISCs/EBs: GFP, green; bGal, white; DI,white). (E) Repressing JNK activity (using Bsk^RNAi^ or Bsk^DN^) in ISC/EBs (using esg^ts^) inhibits ISC over-proliferation induced by loss of Xbp1 or by tunicamycin treatment. Averages and SEM are shown. P values from Student's T test, N = 10. See also Figure S3.

Increased ISC proliferation in Xbp1 or Hrd1 loss of function conditions, or in response to tunicamycin treatment, was associated with increased phosphorylation of JNK in Dl+ ISCs (Figure 5A, Figure S3B), and activation of the JNK target gene *puckered* in all cells of the intestinal epithelium, including ISCs and neighboring ECs (Figure 5B–D, Figure S3A, Figure S3B). This activation can be repressed by over-expressing spliced Xbp1, GTPx-1, or Cat in ISCs, suggesting that JNK is activated in response to ER stress-mediated ROS production (Figure 5D). Since JNK activation in ISCs promotes their proliferative activity [28], we tested whether JNK activity was required for ISC proliferation in Xbp1 loss of function conditions. Indeed, expression of Bsk^RNAi^, or of a dominant-negative version of Bsk (Bsk^DN^), reduced proliferation of ISCs in which Xbp1 was knocked down, and in animals exposed to tunicamycin (Figure 5E). These results suggest that activation of JNK in response to ER-stress-induced ROS production is required in ISCs to induce proliferation.

### Coordinated control of ISC proliferation by the UPR^ER^ and the Keap1/CncC pathway

How do Xbp1 and the UPR^ER^ regulate ISC proliferation? Since promoting ER homeostasis by increasing Xbp1 activity or by stimulating the ERAD pathway was sufficient to limit ISC proliferation in all tested stress and mitogenic conditions, and since the Nrf2 homologue CncC exerts a similar effect on ISC proliferation [19], we asked whether CncC activity was influenced by the ER stress response.

**Figure 6:**
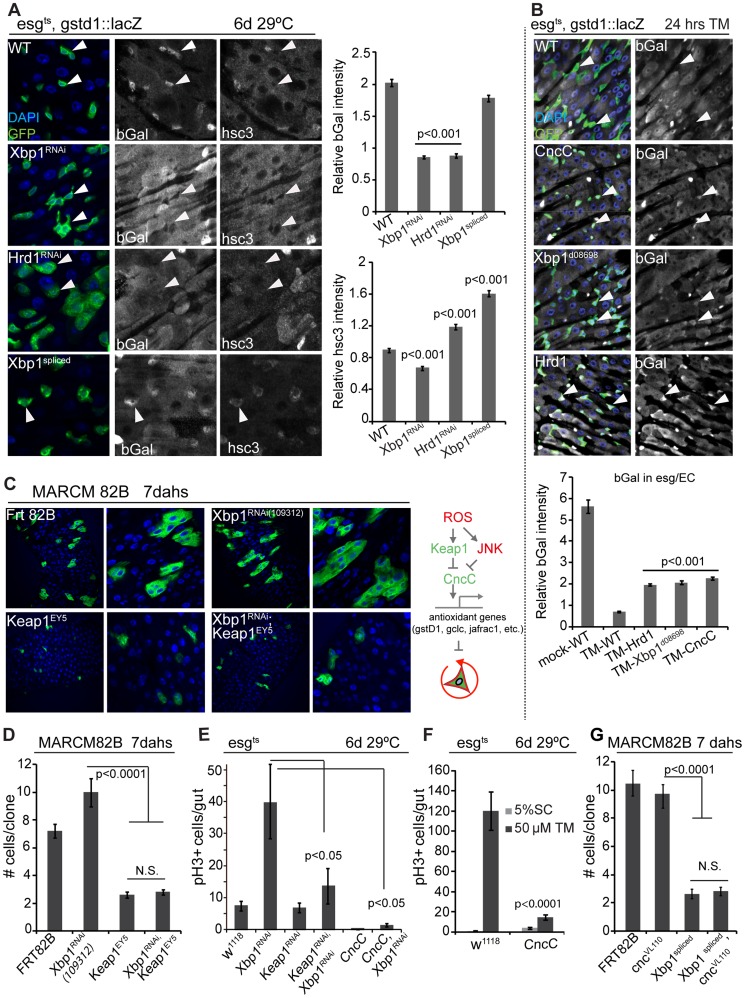
Xbp1 coordinates with Keap-CncC in ISC proliferation. (A) UPR^ER^ influences CncC activity in ISCs/EBs (esgts). CncC activity assessed by bGal expression from the CncC reporter gstd1::lacZ. DAPI, blue; GFP, green; bGal and Hsc3 are shown as separate channels in white. Arrowheads point to individual ISCs/EBs. Quantification of relative bGal and Hsc3 staining shown in the right panel. Ratio of fluorescence intensities in ISCs/EBs and nearby ECs is shown. ISCs/EBs identified by GFP. P values from Student's T test. N = 3. (B) Control of CncC activity by the UPR^ER^ in response to tunicamycin treatment. CncC activity assessed by bGal expression from the CncC reporter gstd1::lacZ. DAPI, blue; GFP, green; bGal white, and shown as separate channel in white. Arrowheads point to individual ISCs/EBs. Quantification of bGal staining shown in the lower panel. Ratio of fluorescence intensities in ISCs/EBs and nearby ECs is shown. ISCs/EBs identified by GFP. P values from Student's T test. N = 3. P values from Student's T test. N = 5. (C) GFP-marked MARCM clones for Keap1^EY5^, Xbp1RNAi^109312^, Keap1^EY5^; Xbp1RNAi^109312^ and wild-type control (Frt82B) at 7 days after heat shock. (D) Quantification of MARCM clone sizes at 7 days after heat shock. Number of clones examined: n = 89 (Frt82B); n = 56 (Xbp1RNAi^109312^); n = 32 (Keap1^EY5^); n = 88 (Keap1^EY5^, Xbp1^RNAi109312^). Averages and SEM are shown. P values from Student's T test. (E) Increased CncC (by loss of Keap1 or over-expressing CncC) inhibits ISC overproliferation induced by loss of Xbp1 in ISCs/EBs (esg^ts^). Averages and SEM are shown. P values from Student's T test. N = 10. (F) CncC in ISCs/EBs (esg::Gal4,tub::Gal80ts) inhibits tunicamycin-induced ISC proliferation. Averages and SEM are shown. P values from Student's T test. N>10. (G) Quantification of MARCM clone sizes for at 7 days after heat shock. Number of clones examined: n = 102 (Frt82B); n = 99 (Xbp1^spliced^); n = 23 (Cnc^VL110^, Xbp1^spliced^). Averages and SEM are shown. P values from Student's T test. See also Figure S4.

To test whether the UPR^ER^ influences CncC activity in ISCs, we used a gstD1::lacZ construct that responds to CncC activity in ISCs [19], [51]. Strikingly, loss of Xbp1 or Hrd1 was sufficient to inhibit gstD1::lacZ expression in ISCs, while ISCs over-expressing spliced Xbp1 maintained high gstD1::lacZ expression (Figure 6A). We confirmed the modulation of Xbp1 activity in these cells by detecting expression of the Xbp1 target *hsc3* (Figure 6A). When ER stress was induced by treating animals with tunicamycin, gstD1::lacZ expression was reduced in ISCs, and this inhibition could be alleviated by over-expressing CncC, Xbp1, or Hrd1 (Figure 6B). The same results were obtained when spliced Xbp1 was expressed (Figure S4A).

Loss of ER homeostasis thus reduces CncC activity in ISCs, suggesting that CncC inhibition is a required component of the ER stress response in the regulation of ISC proliferation. To test this idea, we assessed if ISC proliferation is influenced by the interaction between Xbp1 and CncC. ISCs mutant for the CncC-specific E3 ubiquitin ligase Keap1 do not divide, due to impaired inhibition of CncC activity [19]. ISCs deficient in both Xbp1 and Keap1 did not divide either, suggesting that CncC acts downstream of Xbp1 in the regulation of ISC proliferation (Figure 6C, Figure 6D). Accordingly, knocking down Keap1 or over-expressing CncC was sufficient to rescue ISC over-proliferation caused by loss of Xbp1 (Figure 6E, Figure S4B). Over-expressing CncC was also sufficient to inhibit proliferation induced by tunicamycin treatment (Figure 6F). At the same time, loss of CncC was not sufficient to rescue the proliferation defect of ISCs over-expressing spliced Xbp1, suggesting that Xbp1 inhibits ISC proliferation not only by preventing CncC inhibition, but by additional mechanisms, most likely by inhibiting PERK activation through ER stress (Figure 6G).

### Improved ER quality control in ISCs alleviates age-related intestinal dysplasia

**Figure 7:**
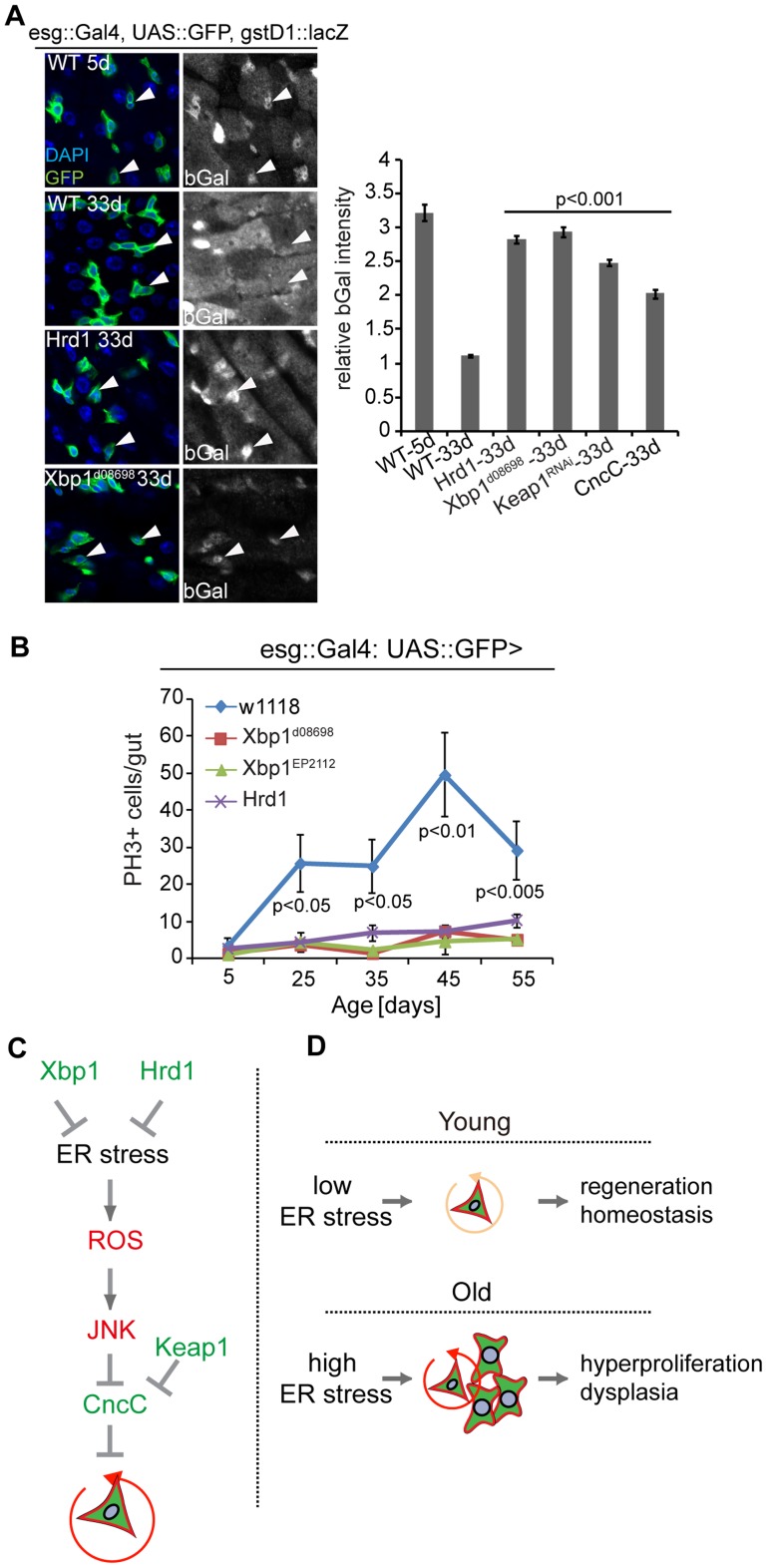
Improved ER quality control alleviates dysplasia of aging ISCs. (A) Intestines of wild-type flies stained with anti-bGal antibodies at 5 days or 33 days of age and of old flies over-expressing Hrd1 or Xbp1 (Xbp1^d08698^) in ISCs/EBs (esg::Gal4) and carrying the gstD1::lacZ reporter. Arrowheads point to ISCs/EBs (marked by GFP). Quantification of relative bGal staining shown in the right panel. Ratio of fluorescence intensity in ISCs/EBs and nearby ECs is shown. ISCs/EBs identified by GFP. P values from Student's T test. N> = 3. (B) Quantification of mitotic figures in aging wild-type flies and in flies expressing Xbp1 (Xbp1^d08698^, Xbp1^EP2112^) and Hrd1 in ISCs/EBs (esg::Gal4, UAS::GFP). Averages and SEM are shown. P values from Student's T test, N>160 (from 4–5 guts each). N>20(5 d), N>20 (25 d), N>25 (35 d), N>7 (45 d), n>14 (55d)) (C) Model of UPR^ER^/ROS signaling network regulating ISC proliferation, through ER stress is required for the induction of ISC proliferation. Inhibition of CncC activity by JNK in response to ER-induced ROS is further required to permit ISC regenerative responses. (D) Age-related loss of proliferative homeostasis as a consequence of increased ER stress. See also Figure S5.

The age-associated activation of the UPR^ER^ in ISCs, and the control of ISC proliferation by the UPR^ER^, suggested that ER stress in ISCs also plays an important role in promoting age-related dysplasia. To address this question, we asked whether promoting ER homeostasis in progenitor cells is sufficient to limit dysplasia. Xbp1 or Hrd1 over-expression was sufficient to maintain expression of gstD1::lacZ, indicating that CncC activity, which declines with age in ISCs [19] was maintained (Figure 7A). Accordingly, Hrd1 expression prevented the age-related increase in *hsc3* expression in ISCs (Figure S5A; CncC or Keap1^RNAi^ expression also preserve gstD1::lacZ expression, confirming the responsiveness of the reporter to CncC activity; Figure S5B). As expected, the same genetic conditions also limit ISC proliferation in aging flies, preventing dysplasia (Figure 7B) [19].

## Discussion

Our results establish a critical role for the coordination of oxidative and ER stress responses in the control of stem cell function, proliferative homeostasis and regenerative capacity in the *Drosophila* intestine. As previously observed for ROS signaling [19], [24], we find that ER stress not only promotes ISC proliferation, but that the UPR^ER^ is also required for ISC proliferation under basal, homeostatic conditions. The UPR^ER^ thus emerges as a rheostat regulating ISC proliferation under both stress and homeostatic conditions. Our results suggest that the tissue-wide increase in ER stress in the aging intestinal epithelium perturbs this regulation, resulting in intestinal dysplasia.

The consequences of perturbing ER homeostasis in the intestinal epithelium are reminiscent of similar effects in Xbp1-deficient mice, where loss of Xbp1 promotes ISC proliferation and intestinal tumorigenesis [2]. At the same time, a recent study suggests that UPR^ER^ components are primarily expressed in transit amplifying cells of the intestinal epithelium, and that activation of the UPR^ER^ (specifically the PERK branch) promotes differentiation of intestinal epithelial stem cells [16]. The *Drosophila* midgut epithelium does not contain a transit amplifying cell population, yet our data suggest that a role for the UPR^ER^ in the control of ISC activity is conserved.

The requirement for CncC inhibition in ER stress-mediated activation of ISC proliferation highlights the integrated control of ISC activity by oxidative and ER stress signals. We propose that Xbp1, by promoting ER homeostasis, limits ROS accumulation in ISCs and thus maintains ISC quiescence (Figure 7D, Figure 7E). Excessive ROS results in JNK activation, which in turn activates Fos and inhibits CncC in ISCs, triggering proliferation ([19], [52] and Li, Hochmuth and Jasper, unpublished results).

This coordination of ER and oxidative stress responses by CncC and the UPR^ER^ is likely to be complex. In *C. elegans* the UPR^ER^ coordinates transcriptional regulation of anti-oxidant genes with the CncC homologue SKN-1 [27]. Interestingly, SKN-1 can also directly control the expression of UPR^ER^ components (including Xbp1, ATF-6 and Bip) by binding to their promoter regions, independent of oxidative stress [27]. Studies in worms have further established the UPR^ER^ as a critical determinant of longevity, and Xbp1 extends lifespan by improving ER stress resistance [53], [54]. Strikingly, local activation of the UPR^ER^ can trigger UPR^ER^ responses in distant tissues, indicating that endocrine processes exist that coordinate such stress responses across cells and tissues [53]–[56]. Our results support the notion that improving proteostasis by boosting ER folding capacity improves long-term tissue homeostasis. These effects seem to be largely mediated by cell-autonomous integration of the UPR^ER^ and redox response by JNK and CncC, but we also observe non-autonomous effects of ER stress on ISC proliferation when knocking down Xbp1 in EBs or ECs selectively. Furthermore, JNK is activated broadly in the intestinal epithelium when Xbp1 or Hrd1 are knocked down in ISCs and EBs, suggesting that non-autonomous interactions between cells experiencing ER stress also influence the regenerative response of this tissue. The molecular events regulating the coordination between cell-autonomous and non-autonomous events in the ER stress response of ISCs are subject of current investigation (Wang et al., in preparation).

In the small intestine of mice, the UPR^ER^ influences regenerative activity not only by influencing ISCs and transit amplifying cells directly, but also by influencing intestinal immune homeostasis. Loss of Xbp1 in intestinal epithelial cells (IECs) leads to apoptosis of secretory Paneth cells and goblet cells, and this pathology is associated with inflammation and higher risk of IBD [1], [3]. Deregulation of innate immune responses by the UPR^ER^ is also found in human patients [1], [3], [57], as well as in *C. elegans* [3], [58]. It can therefore be anticipated that the age-related increase in ER stress in the fly intestine also influences innate immune homeostasis and may contribute to commensal dysbiosis, which we have recently shown to be a driving factor in the age-related loss of proliferative homeostasis of the fly intestine [32]. It will be intriguing to dissect the interaction between the UPR^ER^ machinery, innate immune signaling in ECs, commensal homeostasis and stem cell function in detail, and we anticipate that these interactions have a significant effect on overall lifespan of the organism.

## Materials and Methods

### Fly lines and husbandry

Fly lines w^1118^, frt82B, frt40A, UAS::nlsGFP, UAS::hsc3, UAS::Xbp1^RNAi^ (TRip:HMS03015) were obtained from the Bloomington *Drosophila* stock center. The following RNAi lines were obtained from the Vienna Drosophila RNAi Center: UAS::Xbp1^RNAi^ (v109312, v15347), UAS::Hrd1RNAi (v6870), UAS::bsk^RNAi^.

The following fly lines were generously provided as indicated: *y1w1*; esg::Gal4/+ by Dr. S Hayashi; UAS::xbp1^EP2112^ by Dr. Kyoung Sang Cho; UAS::xbp1^d08698^ by Dr. P. Fernandez-Funez; esg^ts^F/O by Dr. H. Jiang; Su(H)Gbe::Gal4 by Dr. S. Bray; puc^E69^::lacZ by Dr. E. Martín-Blanco; UAS::xbp1^spliced^ by Dr. P. Domingos, UAS::bsk^DN^ by Dr. M. Mlodzik.

The Hrd1 loss of function allele *Hrd1^Delta^* was made by FRT-mediated deletion of sequences between the Pbac insertion lines Pbac{PB}sip3 c00467 and Pbac{PB}faf06363. hs-FLP was expressed in flies in which these Pbac insertions were *in trans*, deleting Hrd1 and its nearby gene CG2126.

All flies were raised on yeast/molasses-based food at 25°C and 65% humidity on a 12 hr light/dark cycle, unless otherwise noted.

For tunicamycin or paraquat exposure, flies were starved in empty vials for 6–8 hrs and fed with a 5% sucrose solution± 50 µM tunicamycin or ±5 mM paraquat for 24 hrs followed by dissection in PBS.

For TARGET experiments, flies were raised at 18°C and shifted to 29°C at certain time points after eclosion. For MARCM clone induction, adult flies were aged for 1–2 days and then heat shocked at 37°C for 45 min.

### Generation of Su (H)GBE-Gal80 transgenic flies

The DNA fragment containing an enhancer of Su(H)GBE and a mini promoter of HSP70 was amplified from Su(H)GBE-Gal4 [39] using PCR, with the following primers:

5′-AGT**GAATTC**AATTAGGCCT**A**CTAGACTTG-3′ (the 20th nucleotide “T” is replaced by “A” to eliminate the endogenous XbaI site).

5′-AGT**TCTAGA**TCATGAT**GCGGCCGC**TCAGGAGGCTTGCTTCAAGCTTG-3′ (a NotI site was introduced in this primer).

The amplified DNA was cut and ligated into EcoRI and XbaI digested pCasper-Tub-Gal80 [1]–[7], [59] to produce the construct pCasper-Su(H)GBE. Then the DNA fragment containing Gal80 and Sv40 polyA was cut from pCasper-Tub-Gal80 at the NotI and XhoI sites, and ligated into NotI- and XhoI-digested pCasper-Su(H)GBE to produce the Su(H)GBE-Gal80 construct.

The construct was sequenced, purified, and microinjected into embryos using the standard method.

### Immunostaining and microscopy

Guts were dissected in PBS, fixed for 45 min at room temperature in 100 mM glutamic acid, 25 mM KCl, 20 mM MgSO_4_, 4 mM sodium phosphate, 1 mM MgCl_2_, and 4%formaldehyde, washed for 1 hr, and incubated with primary antibodies and second antibodies in washing buffer (PBS, 0.5% BSA, 0.1% Triton X-100).

The following primary antibodies were used:, guinea-pig anti-hsc3 antibody antibody [60] (1∶150), mouse anti-Delta (Developmental Studies Hybridoma Bank, 1∶100), rat anti-Delta (gift from Dr. MD Rand, University of Rochester, 1∶1000); rabbit anti-PH3 (phosphorylated histone H3, Upstate, 1∶1000), mouse anti-β-galactosidase (Developmental Studies Hybridoma Bank, 1∶500), rabbit anti-β-galactosidase (Cappel, 1∶5000), rabbit anti-peIF2α antibody (Cell Signaling: 3597, 1∶150), mouse anti-pJNK antibody (Cell Signaling: 9255,1∶150).

For Delta antibody staining, guts were fixed using a methanol-heptane method as descried [61].

Fluorescent secondary antibodies were purchased from Jackson ImmunoResearch Laboratories. DNA was stained using DAPI. Confocal imaging was performed on a Zeiss LSM700 confocal microscope and processed using ImageJ and Adobe Illustrator.

### qRT-PCR analysis of gene expression

Total RNA from young female samples were extracted using Trizol (Invitrogen) and cDNA was synthesized using Superscript III (Invitrogen). Real time RCR was performed on a Bio-Rad CFX96 detection system. Expression Values were normalized to RP49 expression levels. Primers included: total Xbp1 transcripts (Forward:TGGGAGGAGAAAGTGCAAAG, Reverse:TCCGTTCTGTCTGTCAGCTC), Spliced Xbp1 (Forward: ACCAACCTTGGATCTGCCG, Reverse:CGCCAAGCATGTCTTGTAGA), Hrd1 (Forward:GCAGTTGGTCTTTGGCTTTG, Reverse: ATGGGCAGCGCGTATATTT), RP49(Forward:TCCTACCAGCTTCAAGATGAC, Reverse:CACGTTGTGCACCAGGAACT).

### ROS measurement via DHE

ROS levels were measured as described before [19]. Briefly, guts were dissected in Schneider's medium, incubated in 30 µM (Invitrogen) for 5 min at room temperature in the dark, washed twice and mounted to be imaged immediately. GFP expressed under the control of esg::Gal4, Su(H)::Gal80 was used to identify ISCs and/or EBs.

## Supporting Information

Figure S1 The UPR^ER^ is sufficient and required in ISCs to promote proliferation (related to Figure 1, 2, 3). (A) Knockdown Xbp1 or Hrd1 in esg^ts^F/O fly line (using esg::Gal4, tubGal80^ts^, UASFlp, act>STOP>Gal4) accelerates epithelial renewal. Representative images of wild-type fly and fly with loss of Xbp1 are shown on the left. Quantification of clone sizes in the midgut area is shown on the right. (B) Representative images for MARCM clone sizes at 3 days after heat shock for Xbp1 loss-of-function mutant (Xbp1^k13803^) and wild-type control (Frt42D). (C) Representative images for MARCM clone sizes at 3 days after heat shock for knockdown of Xbp1, of Hrd1 and wild-type control (Frt40A). (D) Representative images for MARCM clone sizes at 3 days after heat shock for Hrd1 loss-of-function mutant (Hrd1^Delta^) and wild-type control (Frt82B). (E) Representative images for MARCM clone sizes at 7 days after heat shock for Hrd1 loss-of-function mutant (Hrd1^Delta^) and wild-type control (Frt82B). (F) Representative images for MARCM clone sizes at 7 days after heat shock for spliced Xbp1, Xbp1^d08698^, Hrd1 and wild-type control (Frt82B). (G) ISC-specific knockdown of Xbp1 or Hrd1 (using esg::Gal4, Su(H)-Gbe::G80,tub::Gal80ts) induces ISC proliferation after 4 days of induction (shift to 29°C). Averages and SEM are shown. P values from Student's T test, N>10. (H) Quantification of UPR^ER^ reporters shown in Fig. 1C, 1D, 1E. Relative fluorescence intensity is normalized to wild-type young guts. P values from Student's T test. N = 6 (hsc3 antibody staining); N = 3 (Xbp1p reporter line).
(TIF)

Figure S2 Xbp1s controls ISC proliferation in mitogenic conditions (related to Figure 3). (A) Spliced Xbp1 inhibits Hep(JNKK)-induced ISC over-proliferation in ISCs/EBs (using esg::Gal4, tubG80^ts^).(DNA: DAPI, blue; ISCs/EBs, GFP, ISCs: Delta staining). GFP and Delta channels are shown separately on the right. (B) Quantification of pH3+ cells in guts of 4 days and 14 days expressing Hep or InR or coexpressing Hep or InR with spliced Xbp1in ISCs/EBs (using esg::Gal4, tubG80^ts^). Averages and SEM are shown. P values from Student's Test, N = 10. (C) Deficiency of Notch-induced tumor formation is inhibited by expressed spliced Xbp1, but exacerbated by knockdown Xbp1 in ISCs/EBs (using esg::Gal4, tubG80^ts^). Representative images are shown. (DNA: DAPI blue; ISCs/EBs:GFP, green; ISCs: DI staining; EEs: Prospero staining). GFP, Delta and Prospero channels are shown separately on the right. (D) qRT-PCR validating effectiveness of RNAi and over-expression constructs for UPR^ER^ components. Over-expression of Xbp1 in Xbp1^d08698^ or Xbp1^EP2112^ animals crossed to actin::Gal4 was determined in whole flies. Expression of spliced Xbp1 or Hsc3 using elav::Gal4, as well as knockdown of Xbp1 (Xbp1RNAi^15347^ or Xbp1RNAi^109312^) or Hrd1 using elav::Gal4 was determined in heads. P values from Student's Test, N>3. Expression is relative to *rp49*. (E) Expression of UAS-linked transgenes by esg::Gal4 is limited to Dl+ ISCs by co-expression of Gal80 from the Su(H)Gbe promoter. Arrowheads point to Dl+ ISCs. DAPI blue, GFP green, Dl white.
(TIF)

Figure S3 UPR^ER^-induced ISC proliferation is regulated by JNK activation (related to Figure 5). (A) JNK activation when Xbp1 or Hrd1 is knocked down in ISCs/EBs. Lines expressing dsRNA against Xbp1 (Xbp1RNAi^15347^, Xbp1RNAi^109312^ and Xbp1RNAi^HMS03015^) or Hrd1 were used. puc^E69^::lacZ reporter used to detect JNK activation. bGal is shown as separate channel in white. DAPI, blue; GFP, green; Armadillo, red. (B) Quantification of JNK activity (pJNK antibody and bGal staining for pucE69::lacZ) in Fig. 5A and Fig. S3. Relative fluorescence intensity is normalized to wild-type flies. P values from Student's T test. N = 3. (C) Repressing JNK activity (using Bsk^RNAi^) inhibits ISC over-proliferation induced by loss of Xbp1 in ISC/EBs (using esg^ts^ flip-out). Averages and SEM are shown. P values from Student's T test, N = 10.
(TIF)

Figure S4 Xbp1 regulates ISC proliferation by regulating CncC activity (related to Figure 6). (A) Spliced Xbp1 in ISCs/EBs (using esg::Gal4, UAS-GFP, gstd1::lacZ, tubG80^ts^) maintains high CncC activity in ISCs/EBs under ER stress. (DNA: DAPI blue; ISCs/EBs: GFP). bGal channel is separately shown on the right. (B) Increased CncC activity inhibits ISC overproliferation induced by loss of Xbp1 in esgtsF/O system (esg::Gal4, tubGal80ts, UASFlp, act>STOP>Gal4). Averages and SEM are shown. P values from Student's Test. N = 10.
(TIF)

Figure S5 Effects of Hrd1 and Keap1/CncC on ER stress in aging ISCs (related to Figure 7). (A) Old intestines (50 days) stained with anti-hsc3 antibody for wild-type and fly expressing Hrd1in ISCs/EBs (using esg::Gal4, UAS::GFP). Arrowheads point to the hsc3 staining in ISCs/EBs. (B) Old intestines (33 days) immunostained with anti-bGal and in wild-type fly (same as in Figure 7A) and fly expressing Keap1 loss-of function, or over-expressing CncC. Arrowheads point to individual ISCs/EBs for bGal.
(TIF)
