# Supplementary figures and images for: Integration of UPRER and Oxidative Stress Signaling in the Control of Intestinal Stem Cell Proliferation
Source: PLoS Genet. 2014 Aug 28;10(8):e1004568. doi: 10.1371/journal.pgen.1004568 (PMC4148219; doi:10.1371/journal.pgen.1004568)

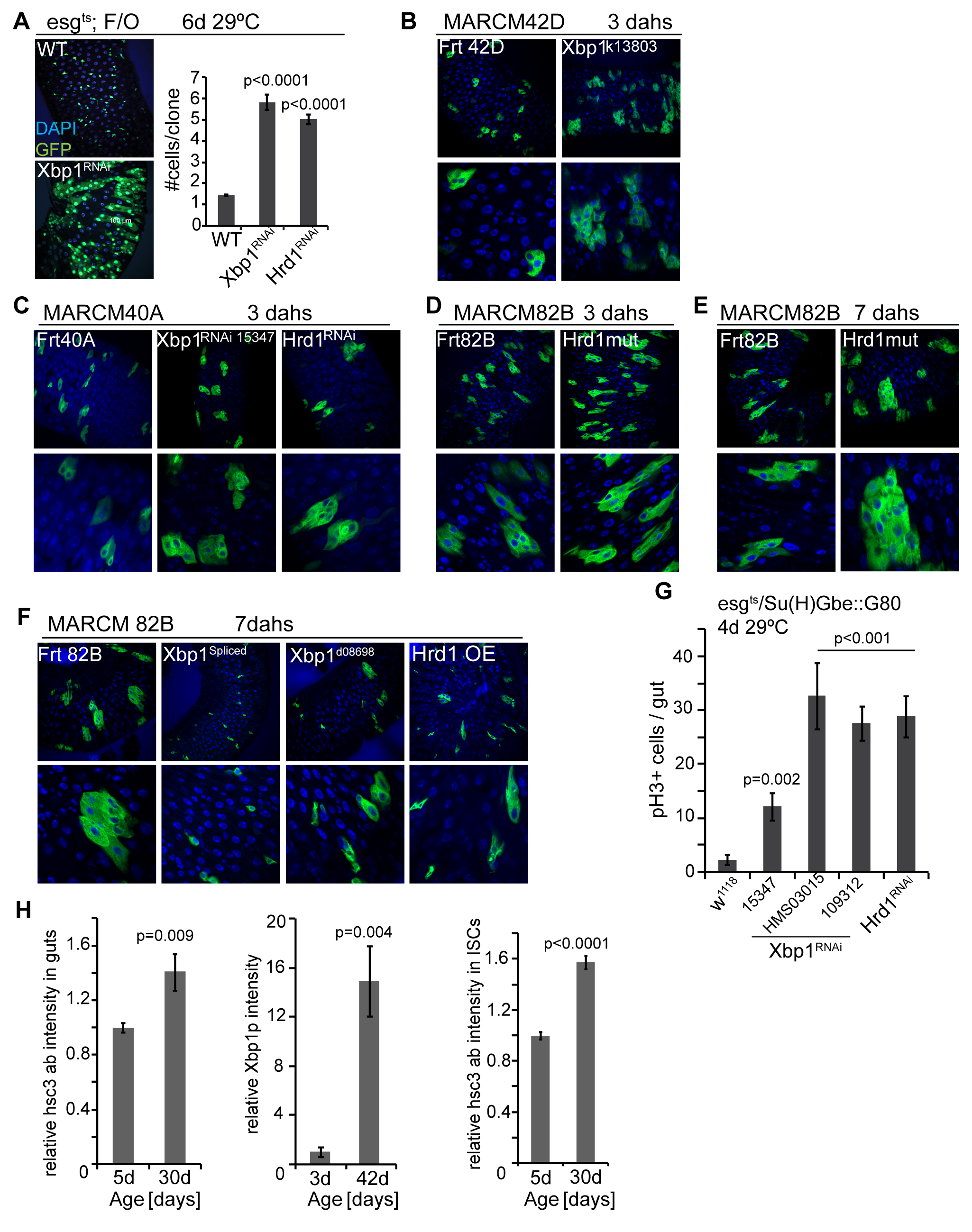

Supplement: Figure S1 — The UPRER is sufficient and required in ISCs to promote proliferation (related to Figure 1, 2, 3). (A) Knockdown Xbp1 or Hrd1 in esgtsF/O fly line (using esg::Gal4, tubGal80ts, UASFlp, act>STOP>Gal4) accelerates epithelial renewal. Representative images of wild-type fly and fly with loss of Xbp1 are shown on the left. Quantification of clone sizes in the midgut area is shown on the right. (B) Representative images for MARCM clone sizes at 3 days after heat shock for Xbp1 loss-of-function mutant (Xbp1k13803) and wild-type control (Frt42D). (C) Representative images for MARCM clone sizes at 3 days after heat shock for knockdown of Xbp1, of Hrd1 and wild-type control (Frt40A). (D) Representative images for MARCM clone sizes at 3 days after heat shock for Hrd1 loss-of-function mutant (Hrd1Delta) and wild-type control (Frt82B). (E) Representative images for MARCM clone sizes at 7 days after heat shock for Hrd1 loss-of-function mutant (Hrd1Delta) and wild-type control (Frt82B). (F) Representative images for MARCM clone sizes at 7 days after heat shock for spliced Xbp1, Xbp1d08698, Hrd1 and wild-type control (Frt82B). (G) ISC-specific knockdown of Xbp1 or Hrd1 (using esg::Gal4, Su(H)-Gbe::G80,tub::Gal80ts) induces ISC proliferation after 4 days of induction (shift to 29°C). Averages and SEM are shown. P values from Student's T test, N>10. (H) Quantification of UPRER reporters shown in Fig. 1C, 1D, 1E. Relative fluorescence intensity is normalized to wild-type young guts. P values from Student's T test. N = 6 (hsc3 antibody staining); N = 3 (Xbp1p reporter line). (TIF) [file pgen.1004568.s001.tif]

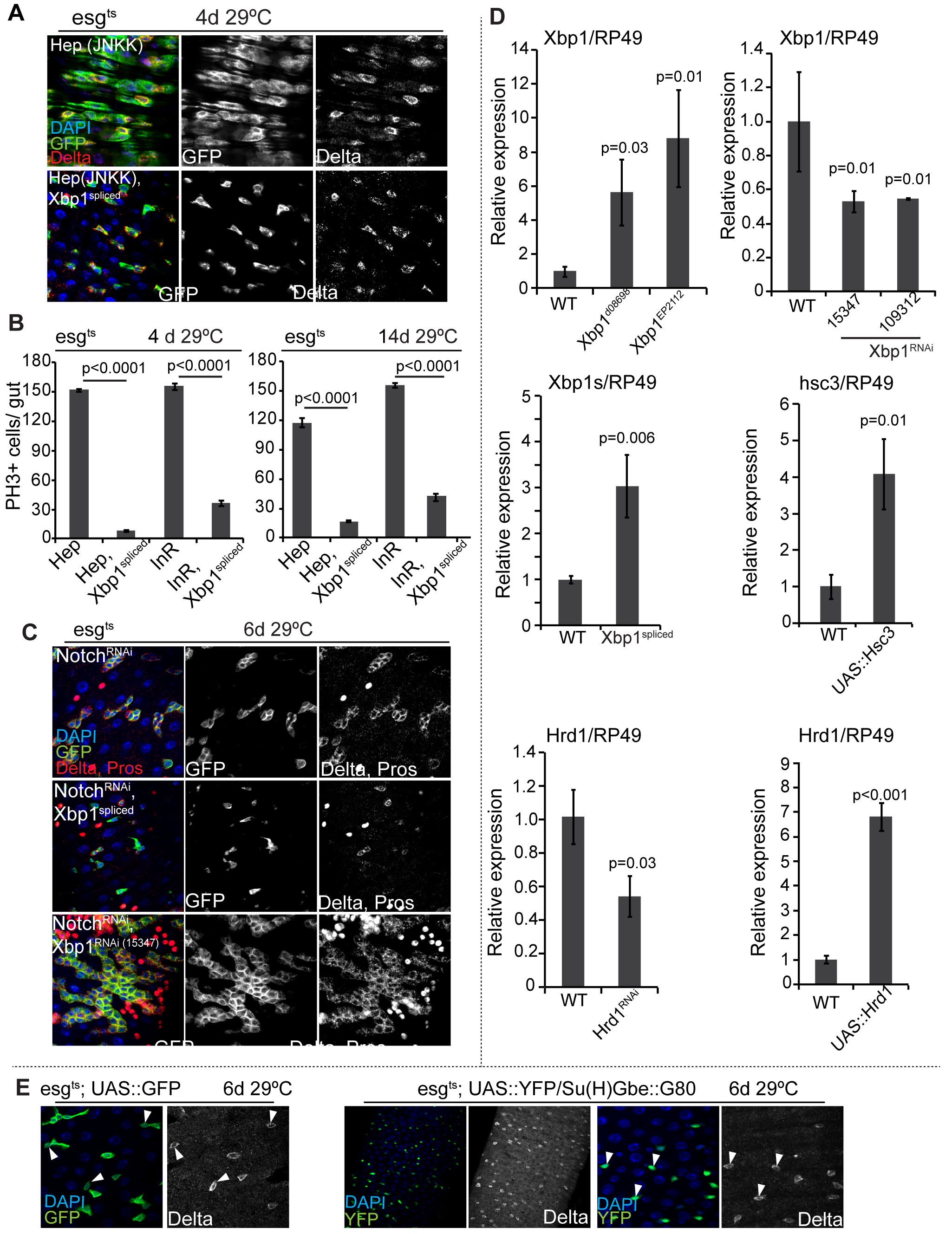

Supplement: Figure S2 — Xbp1s controls ISC proliferation in mitogenic conditions (related to Figure 3). (A) Spliced Xbp1 inhibits Hep(JNKK)-induced ISC over-proliferation in ISCs/EBs (using esg::Gal4, tubG80ts).(DNA: DAPI, blue; ISCs/EBs, GFP, ISCs: Delta staining). GFP and Delta channels are shown separately on the right. (B) Quantification of pH3+ cells in guts of 4 days and 14 days expressing Hep or InR or coexpressing Hep or InR with spliced Xbp1in ISCs/EBs (using esg::Gal4, tubG80ts). Averages and SEM are shown. P values from Student's Test, N = 10. (C) Deficiency of Notch-induced tumor formation is inhibited by expressed spliced Xbp1, but exacerbated by knockdown Xbp1 in ISCs/EBs (using esg::Gal4, tubG80ts). Representative images are shown. (DNA: DAPI blue; ISCs/EBs:GFP, green; ISCs: DI staining; EEs: Prospero staining). GFP, Delta and Prospero channels are shown separately on the right. (D) qRT-PCR validating effectiveness of RNAi and over-expression constructs for UPRER components. Over-expression of Xbp1 in Xbp1d08698 or Xbp1EP2112 animals crossed to actin::Gal4 was determined in whole flies. Expression of spliced Xbp1 or Hsc3 using elav::Gal4, as well as knockdown of Xbp1 (Xbp1RNAi15347 or Xbp1RNAi109312) or Hrd1 using elav::Gal4 was determined in heads. P values from Student's Test, N>3. Expression is relative to rp49. (E) Expression of UAS-linked transgenes by esg::Gal4 is limited to Dl+ ISCs by co-expression of Gal80 from the Su(H)Gbe promoter. Arrowheads point to Dl+ ISCs. DAPI blue, GFP green, Dl white. (TIF) [file pgen.1004568.s002.tif]

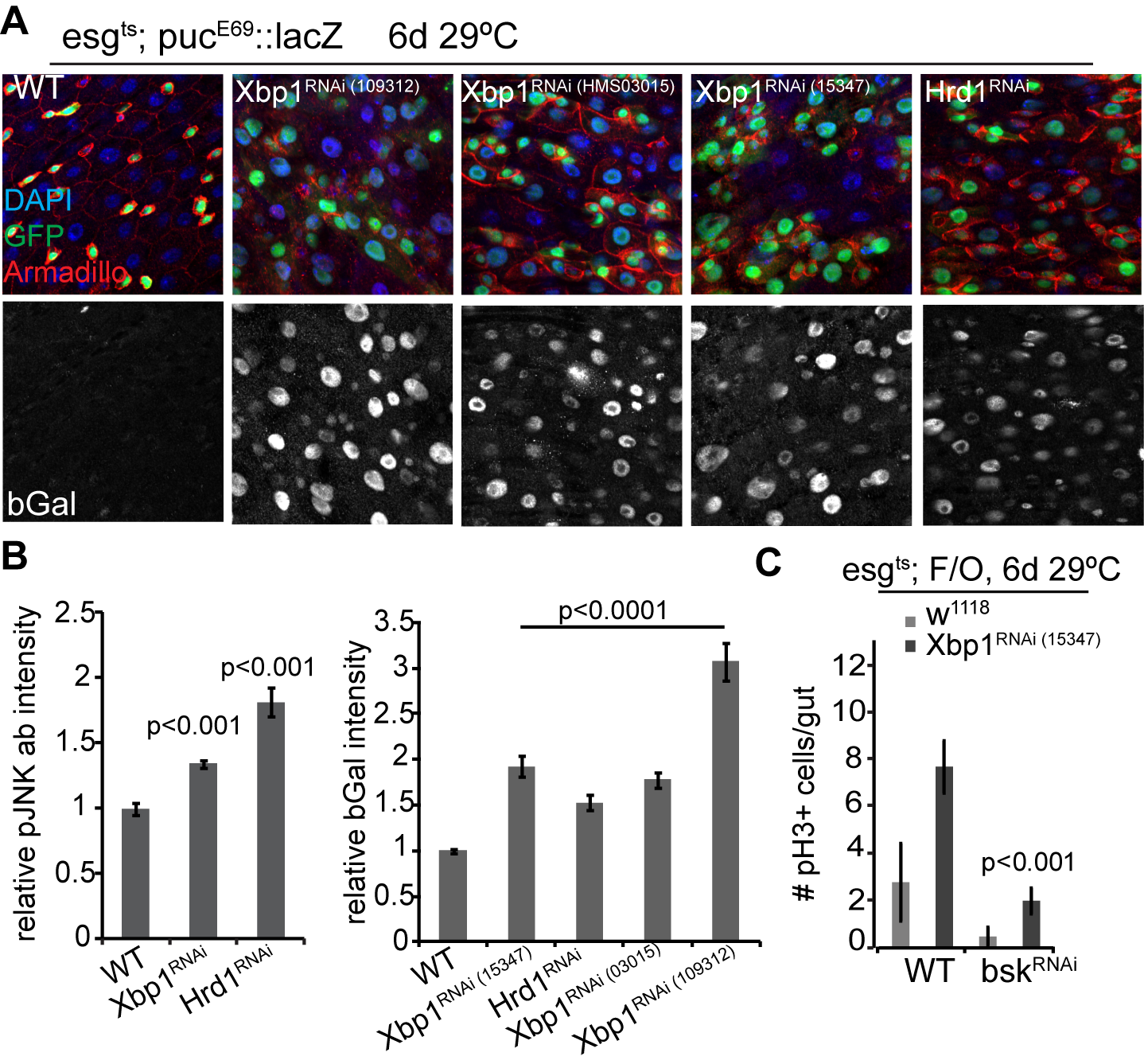

Supplement: Figure S3 — UPRER-induced ISC proliferation is regulated by JNK activation (related to Figure 5). (A) JNK activation when Xbp1 or Hrd1 is knocked down in ISCs/EBs. Lines expressing dsRNA against Xbp1 (Xbp1RNAi15347, Xbp1RNAi109312 and Xbp1RNAiHMS03015) or Hrd1 were used. pucE69::lacZ reporter used to detect JNK activation. bGal is shown as separate channel in white. DAPI, blue; GFP, green; Armadillo, red. (B) Quantification of JNK activity (pJNK antibody and bGal staining for pucE69::lacZ) in Fig. 5A and Fig. S3. Relative fluorescence intensity is normalized to wild-type flies. P values from Student's T test. N = 3. (C) Repressing JNK activity (using BskRNAi) inhibits ISC over-proliferation induced by loss of Xbp1 in ISC/EBs (using esgts flip-out). Averages and SEM are shown. P values from Student's T test, N = 10. (TIF) [file pgen.1004568.s003.tif]

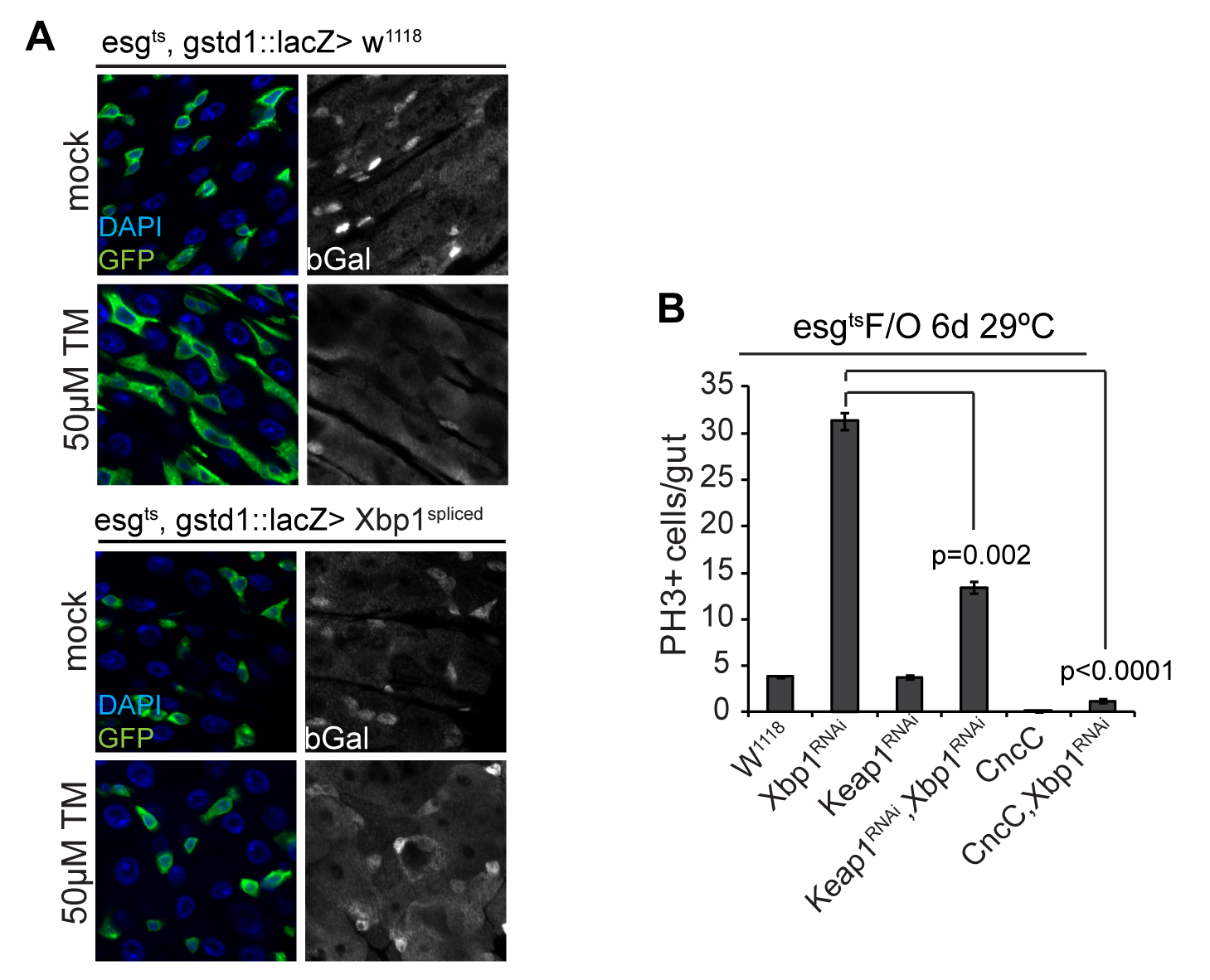

Supplement: Figure S4 — Xbp1 regulates ISC proliferation by regulating CncC activity (related to Figure 6). (A) Spliced Xbp1 in ISCs/EBs (using esg::Gal4, UAS-GFP, gstd1::lacZ, tubG80ts) maintains high CncC activity in ISCs/EBs under ER stress. (DNA: DAPI blue; ISCs/EBs: GFP). bGal channel is separately shown on the right. (B) Increased CncC activity inhibits ISC overproliferation induced by loss of Xbp1 in esgtsF/O system (esg::Gal4, tubGal80ts, UASFlp, act>STOP>Gal4). Averages and SEM are shown. P values from Student's Test. N = 10. (TIF) [file pgen.1004568.s004.tif]

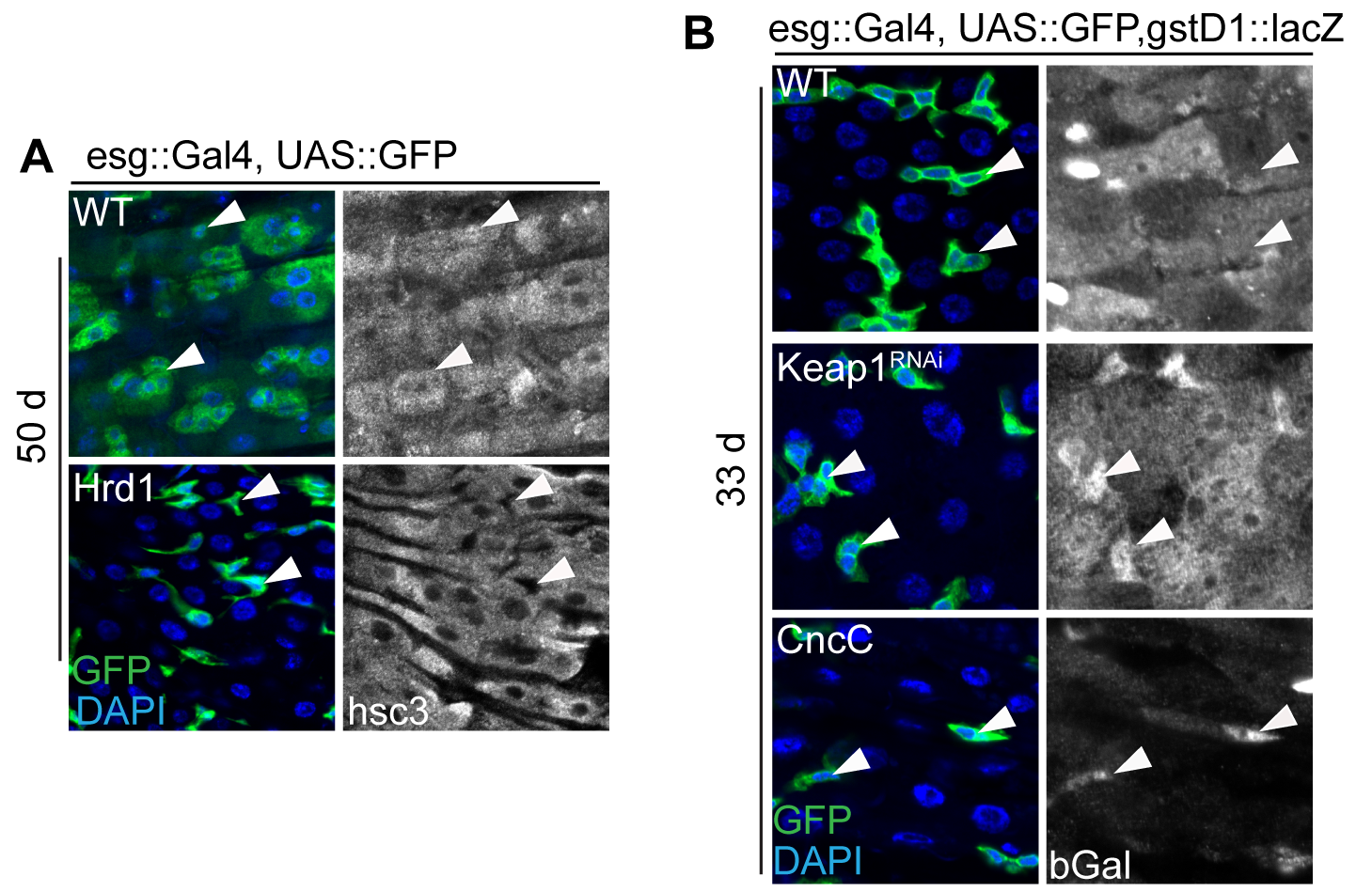

Supplement: Figure S5 — Effects of Hrd1 and Keap1/CncC on ER stress in aging ISCs (related to Figure 7). (A) Old intestines (50 days) stained with anti-hsc3 antibody for wild-type and fly expressing Hrd1in ISCs/EBs (using esg::Gal4, UAS::GFP). Arrowheads point to the hsc3 staining in ISCs/EBs. (B) Old intestines (33 days) immunostained with anti-bGal and in wild-type fly (same as in Figure 7A) and fly expressing Keap1 loss-of function, or over-expressing CncC. Arrowheads point to individual ISCs/EBs for bGal. (TIF) [file pgen.1004568.s005.tif]
